# Glances at the Hospitals

**Published:** 1902-05-10

**Authors:** 


### GLANCES AT THE HOSPITALS.

### THE MEATH HOSPITAL.

THE total ordinary income was £5,272, and the total expenditure was £5,860, showing a deficiency of £588, which, with an adverse balance of £181 19s. in the previous year, makes a total deficiency of about £770. At the beginning of the year there were 112 patients in residence, and 1,334 patients were admitted, making a total of 1,416. Of these 1,260 were discharged cured or relieved, 72 died, and 114 remained under treatment. The average duration of each patient's stay in the hospital was nearly 29 days. The mortality was 5·40 per cent. The daily average number of beds occupied was 105. The usual medical and surgical tables are not given, but there is a *résumé*, from which we extract the following facts: Of enteric fever 79 cases were treated during the year with 4 deaths, showing a mortality of only 5·1 per cent. Of 40 cases of pneumonia 10 died. Of 27 cases of acute rheumatism all recovered. There were 188 operations. Of these cases 171 were discharged cured, 11 were relieved, and 6 died, the rate of mortality after operation being 3·2 per cent. We think it a pity that the usual statistical tables are not given.

### VICTORIA INFIRMARY, GLASGOW.

THE total ordinary income was £7,637 and the total ordinary expenditure was £11,440, showing a deficit on the year's working of £3,800. To meet this deficit the Governors have had to fall back on the capital funds of the institution, and they appeal for more liberal support in the future, remarking that additional buildings are now being erected and that this will entail still further expenditure. Legacies and donations reached the respectable figure of £11,747 during the year. The workpeople's subscriptions amounted to £1,911, which is a fair sum, and there can be no doubt that the rich inhabitants of Glasgow will provide whatever further sum is required to carry on the good work of the infirmary. At the beginning of the year 142 patients were in residence and 1,729 were admitted during the year, making a total of 1,871. Of these 163 died and 145 remained under treatment. The average stay in the hospital was 29 days. We notice that 25 cases of acute rheumatism were treated and that all of them recovered. Of 47 cases of pneumonia 39 recovered and 8 died. There were 100 cases of abdominal section for appendicitis, gastric ulcer, malignant disease, ovarian tumour, etc., and of these 75 recovered and 25 died. The operation for the radical cure of hernia was performed 23 times and all were successful. Nephrectomy was performed 4 times, all being successful. The total number of operations was 778, and the results show that 724 were followed by cure or relief and 54 by death. We miss the table showing the nature of the anæsthetic used.

### WEST BROMWICH DISTRICT HOSPITAL.

THE income was £3,699, and the expenditure was £3,647. The income was £200 in excess of that of last year, and the governors call attention to the improvement in the finances, and they state that the deficiency which had accumulated in previous years has been reduced. An instructive table is given at page 20 of the report. It shows that the hospital was opened in 1867. In 1871 there were 64 in-patients and 1,223 out-patients. The total sum of money received was £773. In the year 1900 the number of in-patients was 720, and the out-patients 8,504, while the total sum of money received had risen to £3,698. Last year the cost per bed occupied was £59 14s., the average stay in the hospital was 22 days, and the death-rate was 8·3 per cent. The medical and surgical tables do not show results, but merely give the numbers treated for the various diseases, hence these tables are of but little use. A mortality table, however, gives the cause of death in each case. A total of 223 operations were performed during the year, but the anæsthetic used is not mentioned.

### ROYAL EYE HOSPITAL, MANCHESTER.

AT this hospital the income and expenditure almost balanced each other. The income was £4,800, and the expenditure exceeded it by £19. It is stated that the hospital has continued its career of usefulness, and has surpassed all previous years in the number of patients treated. The hospital is a very old one, having been founded in 1814. In 1864 it had 3,205 out-patients, and 215 in-patients; the income being a little over £1,000 a year. Last year it had 24,135 out-patients, 1,356 in-patients; 5,062 accidents were attended to, and 1,976 operations were performed. The number of beds in the hospital is 110, the average number occupied was 79, the average stay in the hospital was 21 days, and the cost of each patient's board was 4s. 2d. a week. The cataract cases are very carefully detailed, and the following is the summary of the 227 cases. In 206 the operation was successful, and the resulting vision good, in 5 it was partially successful, in 7 the operation was successful, but the vision defective from diseased state of the eye before operation, in 2 the eye was lost, and in 7 the vision was not recorded. Von Graefe's linear method was employed in 210 of the cases. In all cases the anæsthetic used was a 4 per cent. solution of the hydrochlorate of cocaine, and in no case could any ill effects be attributed to it.

### BOSTON CITY HOSPITAL.

THE entire expenditure at this hospital for the year amounted to upwards of 433,000 dollars, but only 302,000 dollars were expended on the hospital proper, and this again has to be reduced to about 294,000 dollars as the stock in hand was greater than in the previous year. The weekly cost per patient was 13½ dollars. At the beginning of the year the hospital contained 447, and 8,131 were admitted during the year, making a total of 8,578 under treatment. The highest number in the hospital at any one time was 495, and the daily average number was 418. The average stay in the hospital was 17 days. In the out-patients' department 27,328 patients were treated, and the total number of attendances was 99,125, the maintenance cost 9,396 dollars. Turning to the medical and surgical tables, which are unusually clear, we find that 391 cases of enteric fever were treated. Of these 317 were cured, 23 relieved, 5 not relieved, 1 not treated, 45 died, and 34 remained under treatment. The operation for appendicitis was performed 173 times. Of these 27 died and 20 remained under treatment.

### HULL ROYAL INFIRMARY.

THE ordinary receipts amounted to £9,774, and the expenditure exceeded the income by £319. The workpeople's donations amounted to £2,028. The total number of in-patients was 2,638. The average number of beds occupied was 165, being 5 above that of the previous year, and the average stay in the hospital of each patient was nearly 23 days. The average cost of each in-patient was £2 16s. 9d., and the cost per head per week was 17s. 4d. The cost per bed occupied was £50 15s. The total number of out-patients was 14,574, involving a total of 76,980 attendances. The estimated cost of each out-patient was 1s. 4½d. It is proposed to enlarge the Infirmary at a cost of nearly £50,000. No medical or surgical statistics are given.

### BRADFORD ROYAL INFIRMARY.

THE governors say that the finances of the infirmary have become a matter of very serious concern to those who are responsible for their management; and they prove this by pointing out that there is an adverse balance of £3,918. It is evident that either the income must be materially increased or the Board must reduce the expenditure; and the latter can only be effected by closing a number of beds. Even then the saving is not so great as it might appear to the non-initiated as the establishment charges go on almost the same whether the average number in the infirmary be 160 or 120. It is only the patients' maintenance that is saved. Bradford is a large town and a rich one, and when it is realised that more subscriptions must be forthcoming to insure the continuance of the usefulness of the hospital, we believe that the necessary funds will be forthcoming. In the meantime it would be desirable to see whether the workpeople's own donations could not be largely increased. The income from all sources amounted to £7,500; and the expenditure to £11,358, but this included a sum of £1,135 due to the bank. The medical and surgical tables give all necessary information, except that it might be advisable in future reports to divide the column for "discharged" into "cured" and "relieved." For instance, we notice among the surgical tables that there were under treatment 30 cases of ovarian and tubal disease. Of these 3 died, 4 remained in the hospital, and 23 were discharged, but whether "cured" or "relieved" we do not know. Thirty-three amputations were performed with only one death. The operation for strangulated hernia was performed 16 times. Of these 11 recovered and 5 died. For the radical cure of hernia the operation was performed 22 times with one death. Of 24 cases of acute rheumatism 23 recovered and one remained in the hospital. The total number of in-cases was 700. Of these 424 were cured or relieved, 78 not relieved, and 81 died. The death-rate was 11·85 per cent., or, deducting 7 deaths occurring within 24 hours of admission, 10·85 per cent.

